# Sex-based differences in outcomes after severe injury: an analysis of blunt trauma patients in China

**DOI:** 10.1186/s13049-017-0389-6

**Published:** 2017-05-02

**Authors:** Ziqiang Zhu, Xiaoping Shang, Peiyi Qi, Shengli Ma

**Affiliations:** 1 Department of Emergency, The First Affiliated Hospital of Zhengzhou University, No.1 Jian She Dong Avenue, Zhengzhou, 450002 People’s Republic of China; 2 Department of Medical Records, The First Affiliated Hospital of Zhengzhou University, No.1 Jian She Dong Avenue, Zhengzhou, 450002 People’s Republic of China

**Keywords:** Blunt trauma, Complications, Sex, Estrogen, Mortality

## Abstract

**Background:**

Experimental research suggests that females have a higher survival rate after trauma, although this claim is controversial. This study sought to determine the role of sex on mortality among trauma patients in China.

**Methods:**

The study enrolled 1789 trauma patients who visited the Emergency Intensive Care Unit of the First Affiliated Hospital of Zhengzhou University during 2015 and 2016. A retrospective data analysis was performed to determine sex-based differences after blunt trauma. Patients were stratified by age and injury severity (using the Injury Severity Score). Multiple logistic regression was used to analyze the association between sex and post-injury complications and mortality.

**Results:**

Female trauma patients experienced a significantly lower risk of mortality than males (odds ratio, 0.931; 95% confidence interval, 0.883–0.982). This survival advantage of females was particularly notable in the ‘younger than 45 years’ age group. Sex-based differences were also found in the occurrence of life-threatening complications after trauma.

**Conclusion:**

This study demonstrated that females are more likely to survival after severe blunt trauma and also have less inpatient complications than men, suggesting an important role for sex hormones after severe traumatic injury.

## Background

There is an increasing amount of evidence to suggest sexual dimorphism in response to traumatic injury, with a survival advantage to be found in females compared to males [1–6]. The primary sex hormones, estrogen and testosterone, have been shown to regulate immunity by altering the T helper (Th)-1 and Th-2 profiles in cell-mediated immune function [7], as well as influencing the synthesis and release of cytokines (such as interleukin (IL)-1 and IL-6) [8, 9]. Many animal studies collectively suggest that estrogen protects immune cell effector responses [10, 11]. Conversely, male sex hormones were reported to exhibit suppressive effects on immunity [12].

In addition to improved immune responses, female hormones were also suggested to be responsible for improving or maintaining the functions of organs such as the heart, liver, lung, and intestines following trauma-hemorrhage [13–16]. Furthermore, several of these studies indicate that this protection is related to the hormonal status of females, i.e., resistance to trauma hemorrhage-induced organ injury is enhanced as estrogen levels increase, and maximal protection is conferred during the proestrus and estrus stages of the estrous cycle [17, 18].

Although previous animal studies have demonstrated the advantages of the female sex and younger age, clinical studies of trauma outcomes in men and women have produced mixed results. Some reports have suggested that women have a lower overall mortality after trauma [19–24], while other studies concluded that there is no such sex-based difference in outcomes [25–28]. Recently, racial disparities have also been investigated in post-injury functional outcomes. A study conducted by Sperry et al. [29] in the United States revealed that Caucasian females had a lower risk of mortality after severe injury, while no significant sex dimorphism was found for Hispanics or blacks. They also found that Asians descent was shown to have an exaggerated sexual dimorphism for the risk of mortality as compared with whites, blacks, and Hispanics.

Due to the findings of sexual dimorphism on trauma outcome in humans are less and controversial, so more research is needed to address this point. This present study herein sought to investigate the effect of gender on outcome differences (mortality, inpatient complications, ventilator usage, length of hospital stay, and so on) in Chinese trauma patients stratified for age. The study also seeks to understand if female sex is associated with the risk of mortality and inpatient complications among Chinese trauma patients.

## Methods

### Subjects

All trauma patients between the ages of 13 and 65 years who were admitted to the Emergency Intensive Care Unit of the First Affiliated Hospital of Zhengzhou University Acute Trauma Center between January 2015 and May 2016 were included in this study. In order to eliminate the influence of racial disparities, the objects of this study are composed of only Han Chinese. Injury severity score (ISS) was measured to assess the injury severity of patients when they were sent to our hospital by an emergency specialist. Pregnant women and those with an ISS less than 16 points were excluded from this study. Additionally, patients who survived no more than 24 h from the time of injury were excluded, as were patients with a history of steroid use or hormone replacement therapy. In patients with penetrating injuries, factors that can affect the risk of mortality include not only the severity of injuries, but also the trajectory of the force causing the injury. Therefore, we studied only blunt trauma patients. The Ethical Committee of the Zhengzhou University, Henan, China, approved the study, and informed consent was obtained from all patients for this study.

### Data collection and outcome measures

In this study, selected patients were stratified based on age in an attempt to elucidate the protective effect of estrogens in adolescent girls and younger women (13–45 years old), women in their perimenopausal period (45–50 years old), and women in their postmenopausal period (≥50 years old). Patients were also divided into two groups by ISS (16 ≤ ISS < 25 vs. ISS ≥25). Demographic and injury severity characteristics such as sex, age, ISS, presenting systolic blood pressure (SBP), presenting Glasgow Coma Scale (GCS), base excess, presence of hypotension on arrival (SBP, ≤90 mmHg), blood transfusion, the need for emergency surgery intervention, and intubation status were compared for men and women. Hospital resource use was evaluated by ventilator-use days, intensive care unit (ICU) days, and hospital days. In addition, different complication after traumatic injury such as pneumonia, acute renal failure (ARF), pulmonary embolism (PE), acute respiratory distress syndrome (ARDS), and intra-abdominal abscess (IAA) were also analyzed in this study. These complications were selected according to Chinese trauma database, and they were considered as most common and potentially life-threatening complications.

### Statistical analyses

SPSS (version 17.0) was used for statistical analysis. The continuous variables were expressed as mean ± SD, and Student’s t tests were used to compare means between the two groups. The qualitative variables were expressed as proportions and were compared using the chi-squared test. Multiple logistic regression analysis was applied to identify significant predictive factors for mortality, and regression models were adjusted for possible confounding factors including age, hypotension (SBP ≤90 mmHg), ISS, presenting GCS, and time of transfer from the emergency department to the operating room. The logistic regression analysis results are presented as odds ratios (ORs) with 95% confidence intervals (CI). Unless otherwise stated, *P* < 0.05 was determined to be significant. Statistical analyses were performed using SPSS 19.0 (IBM SPSS).

## Results

### Patient characteristics

A total of 1789 blunt trauma patients were included in this study, including 1367 male and 422 female patients. Characteristics of the study patients are shown in Table 1. The mean age was 45.1 ± 17.4 years for males and 46.2 ± 15.9 years for females. All the patients had significant injures, with a mean ISS of 23.6 ± 8.7, and the mean blood transfusion within the first 24 h postinjury were 2.9 ± 2.5 units of packed red blood cells. 16.3% and 14.7% of male and female patients required emergency surgery, respectively. The presenting SBP for males and females were (125 ± 21) mmHg and (119 ± 23) mmHg, respectively; 21.7% of male and 20.3% of female patients were found to be hypotensive upon their arrival in the emergency room. There were no statistical differences in age, presenting GCS score, intubation status, and blood transfusion between the sexes (Table 1).

**Table 1:** Injury characteristics of the study population by gender

Variables	Male (*n* = 1367)	Female (*n* = 422)	*P* value
Age, years	45.1 ± 17.4	46.2 ± 15.9	0.247
Admission SBP, mmHg	123 ± 24	121 ± 28	0.151
Hypotension (SBP ≤ 90 mmHg), n (%)	297 (21.7%)	86 (20.3%)	0.555
ISS	23.6 ± 8.2	23.5 ± 7.3	0.822
Admission GCS score	14.3 ± 3.8	14.6 ± 2.6	0.130
Blood transfusion (24 h), units	2.9 ± 2.3	2.8 ± 2.1	0.426
ED to OR, n (%)	223 (16.3%)	62 (14.7%)	0.426
Intubation status (% yes)	38 (2.8%)	13 (3.1%)	0.746

*GCS* Glasgow Coma Scale, *ISS* Injury Severity Score, *ED* Emergency department, *OR* Operating room, *SBP* Systolic blood pressure

On examining the association between sex and post-injury complications, it was observed that women had a lower prevalence of all complications studied, including pneumonia, acute respiratory distress syndrome, acute renal failure, pulmonary embolism, and intra-abdominal abscess (Table 2). Women were also found to have lower odds of developing each of these complications compared to men (Table 2).

**Table 2:** Adjusted odds of death for patients who developed any of the listed major complications

Major complication	Male (*n* = 1367)	Female (*n* = 422)	OR (95% CI) female vs. male
Pneumonia	3.96	2.63	0.61 (0.53–0.70)
Acute respiratory distress syndrome	1.64	1.27	0.75 (0.70–0.81)
Acute renal failure	0.82	0.54	0.63 (0.55–0.72)
Pulmonary embolism	0.53	0.31	0.42 (0.34–0.51)
Intra-abdominal abscess	0.26	0.13	0.78 (0.73–0.83)

*OR* odds ratio, *CI* confidence interval

### Comparison of outcomes by sex, age, and injury severity

Patients were then analyzed by three age groups: ages 13– 45, 45–50, and older than 50 years (Table 3). Clinical outcomes were compared between the sexes in each group. In the 13–45 years age group, there was no difference in hospital resource use. A ventilator was more frequently used by men, and women had significantly lower mortalities than men. In the older than 50 years age group, ventilator use was also found to be more frequent in men. However, women had a longer length of stay in the ICU than men in both the 45–50 years age group and the older than 50 years group; there is no difference in mortality between the two sexes.

**Table 3:** Outcome comparisons by sex and age groups

Variables	13 ≤ Age < 45			45 ≤ Age < 50			Age ≥50		
	Male	Female	*P* value	Male	Female	*P* value	Male	Female	*P* value
n	796	263	-	82	30	-	489	129	-
Ventilator, %	92 (11.6%)	14 (5.3%)	0.003	10 (12.1%)	2 (6.7%)	0.402	60 (12.7%)	8 (6.2%)	0.039
Duration ventilation, days	10.2 ± 9.8	9.1 ± 7.9	0.099	9.6 ± 10.3	8.7 ± 8.9	0.673	8.3 ± 91	7.2 ± 8.7	0.218
Days at ICU	10.6 ± 4.6	10.2 ± 5.2	0.237	8.1 ± 5.4	12.8 ± 5.1	<0.001	11.5 ± 4.3	14.2 ± 5.9	<0.001
Length of hospital stay, days	21.3 ± 11.3	23.5 ± 10.4	0.128	20.3 ± 10.9	18.9 ± 11.8	0.557	21.1 ± 15.1	23.5 ± 10.9	0.091
Mortality, n (%)	66 (8.3%)	6 (3.7%)	0.001	5 (6.1%)	2 (6.7%)	0.912	54 (11.0%)	8 (6.2%)	0.104

ICU, Intensive Care Unit

Trauma patients in each age group were divided according to their ISS into moderate injury (16 ≤ ISS <25) and severe injury (ISS ≥25) groups. Outcomes are shown in Table 4. Both moderately and severely injured patients in the 13–45 years age group had similar characteristics between sexes, although women had a significantly lower mortality and frequency of ventilator use than men. In the 45–50 years age group, male patients in the 16 ≤ ISS < 25 group had a significantly higher ventilator use rate and longer ICU stay. In the older than 50 years age group and 16 ≤ ISS < 25 group, no difference was found between sexes. As for patients older than 50 years and ISS ≥25, ventilator use and mortality were higher in men, while women had longer ICU stays.

**Table 4:** Outcomes comparison by age group and injury severity

Variables	13 ≤ Age < 45						45 ≤ Age < 50						Age ≥50					
	16 ≤ ISS < 25			ISS ≥25			16 ≤ ISS < 25			ISS ≥25			16 ≤ ISS < 25			ISS ≥25		
	Male	Female	*P*	Male	Female	*P*	Male	Female	*P*	Male	Female	*P*	Male	Female	*P*	Male	Female	*P*
n	583	155	-	213	108		57	18		25	12		357	80		132	49	
Ventilator, %	51 (8.7%)	5 (3.2%)	0.021	41 (19.2%)	10 (9.3%)	0.021	5 (8.8%)	1 (3.0%)	0.623	5 (20.0%)	1 (12.3%)	0.367	27 (7.6%)	3 (3.8%)	0.223	33 (24.6%)	5 (10.2%)	0.03
Duration of ventilation, days	6.8 ± 5.2	6.6 ± 6.1	0.682	10.8 ± 7.1	9.7 ± 6.3	0.175	6.2 ± 5.0	5.8 ± 7.2	0.792	10.6 ± 7.4	9.1 ± 6.3	0.550	6.7 ± 4.8	6.1 ± 5.3	0.322	10.1 ± 6.7	8.4 ± 5.9	0.12
Days at ICU	9.3 ± 3.7	8.8 ± 4.6	0.157	11.1 ± 8.2	9.3 ± 8.9	0.072	7.6 ± 4.3	11.4 ± 3.8	0.001	9.6 ± 7.6	13.5 ± 9.3	0.183	11.2 ± 4.5	12.0 ± 3.9	0.142	12.3 ± 7.8	16.1 ± 9.0	0.006
Length of hospital stay, days	21.3 ± 11.3	22.6 ± 10.6	0.198	23.1 ± 12.5	24.3 ± 13.2	0.426	19.4 ± 12.3	18.1 ± 15.6	0.716	21.2 ± 14.9	19.3 ± 15.6	0.723	19.8 ± 13.8	23.1 ± 14.3	0.056	21.5 ± 16.3	25.8 ± 15.1	0.11
Mortality, n(%)	36 (6.2%)	3 (1.9%)	0.036	30 (14.1%)	3 (2.8%)	0.002	2 (3.5%)	1 (5.6%)	0.828	3 (12.0%)	1 (8.3%)	0.737	26 (7.3%)	4 (5.0%)	0.465	28 (21.2%)	4 (8.2%)	0.041

*ISS* Injury Severity Score, *ICU* Intensive Care Unit

### Potential risk factors for mortality

The logistic regression models for mortality were developed based on information in the database, and the independent variables included sex, admission Glasgow Coma Scale (CGS) score, ISS, age and blood transfusion within 24 h. The unadjusted and adjusted odds ratios are shown in Table 5. The adjusted model revealed that sex was an independent risk factor for mortality, with the adjusted OR of morbidity 0.931 times lower in females compared to male patients. Moreover, we found that the mortality was significantly increased with age. The adjusted OR of morbidity in the 45–50 years and older than 50 years age groups were 1.928 and 4.836 times higher than that of the 13–45 years age group, respectively. ISS was also found to be an independent risk factor for mortality, while the higher admission CGS score was a protective factor for mortality. Blood transfusion with 24 h is not the risk factor for mortality.

**Table 5:** Unadjusted and adjusted odds ratios for overall mortality

Variable	Unadjusted model		Adjusted model	
	OR	95% CI	OR	95% CI
Gender				
Male (reference)	1		1	
Female	0.832	0.715–0.969	0.931	0.883–0.982
Admission GCS	0.635	0.423–0.953	0.593	0.424–0.829
ISS	1.231	1.024–1.480	1.145	1.074–1.220
Age				
13–45 (reference)	1		1	
45–50	1.534	0.839–2.804	1.928	0.954–3.895
≥ 50	3.687	2.015–6.747	4.836	2.160–10.829
Blood transfusion				
< 2 unit	1		1	
2–4 unit	1.124	0.867–1.214	1.214	0.906–1.521
> 4 unit	1.421	0.912–1.667	1.503	0.942–1.928

*GCS* Glasgow Coma Scale, *ISS* Injury Severity Score, *OR* odds ratio, *CI* confidence interval. Confounding variables controlled in regression models include age, ISS, presenting GCS, hypotension (SBP ≤ 90 mmHg), Admission GCS score, intubation status, and the requirement of emergency operative intervention

## Discussion

In this study, we found that male sex was associated with an increased incidence of mortality. This result is consistent with the findings of some previous clinical studies [20, 21, 24]. However, there are some other studies that indicate no substantial differences between males and females after blunt trauma [25–28]. A study conducted by Sperry et al. [29] investigated racial differences after severe injury, and they found that Asians exhibit the largest sex-based differences in mortality compared with Caucasians, Hispanics or blacks. Thus, ethnicity appears likely to contribute to the variability of sex-based mortality differences. A subsequent clinical study conducted in China showed similar results [24], with an even more exaggerated sexual dimorphism (a 62% lower mortality in females) in an Asian population. In our study, we also observed lower mortality in females, which is further evidence of Asians exhibiting sex-based outcome disparities after traumatic injury.

Our findings also implicate hormonal differences in sex-based outcome disparities. Previous studies have already demonstrated that the difference in outcome after trauma appears to be related to both testosterone and estrogen. Estrogen has been shown to have an immune-enhancing effect that improves survival after trauma. Administration of estrogen reversed the decrease in T- and B-cell function, restored splenic dendritic cell and macrophage antigen presentation functions, and maintained cytokine release [9, 30–32]. Meanwhile, testosterone is thought to have a deleterious effect on survival [33, 34]. Studies also showed that only animals in the proestrus stage experienced a protective effect against mortality after trauma-hemorrhage [30, 35], because estrogen levels in this phase of the estrous cycle are at their peak. A retrospective analysis conducted by Haider et al. showed that adolescent females in hormonally active ages are less likely to die after trauma when compared to equivalently-injured and age matched males [36]. The present study also found that only females younger than 45 years had a statistically significant survival advantage over males of the same age group; women older than 45 did not have a survival advantage. Since women begin to undergo menopause around the age of 45, it follows that women lose their survival advantage when they are 45 years of age or older. The above findings support the hypothesis that sex hormones play a role in increased survival among women after trauma.

This study also demonstrated that males are at greater risk of developing certain life-threatening complications, including pneumonia, acute respiratory distress syndrome, acute renal failure, pulmonary embolism, and intra-abdominal abscess. This finding is consistent with a previous study that showed that women have fewer complications following trauma compared to similarly injured men [37]. Our data provide additional evidence to support previous reports of improved survival among females after trauma, and the protective benefit in women most likely involves a reduced susceptibility to developing complications. However, previous studies also found that women with any life-threatening complication had somewhat higher adjusted odds of death when they were compared to equivalently injured men with the same complications, meaning that once these women acquired a life-threatening complication, they are more likely to die [27, 37, 38]. It is still not clear why women have a lower rate of complications but a higher associated mortality than men. Due to clinical resource constraints in our study, we did not investigate the risk of death after the individuals developed complications; hence, additional clinical studies are required to clarify this phenomenon.

The present study also demonstrates that in the group of age ≥ 50, sexual dimorphism was found in patients with an ISS of 25 or higher and not in patients with an ISS less than 25. While in the other age groups, sexual dimorphism was not related to ISS. Previous studies showed that protection of female trauma patients against mortality was strongest in those with severe injury, and they speculate that sex-based outcome differences may be more obvious in severe blunt trauma patients [24, 29]. However, in our study, we believe that this inference applies only to a certain age group (≥50 years old).

Our study has some limitations. First, this study included only 1789 patients in China. Therefore, the limited number of individuals included in the subgroup analysis may have led to skewing of the results. Second, since this is a retrospective study, we assumed that younger women up to 45 years of age are premenopausal and those older than 50 are postmenopausal. However, we could not confirm whether older female patients were actually menopausal. Obtaining details of the actual menopausal state of our female patients would have made the results more accurate. Third, some other factors such as financial situations, medical insurance, smoking and drinking history, and illicit drug use are all likely to impact outcomes after trauma. We did not investigate these variables in the present study; future studies ought to address such factors.

## Conclusion

The current study showed sexual dimorphism in Chinese patients regarding outcomes after blunt trauma; this, combined with previous studies, support the possibility of race being a factor in the outcome after injury. Moreover, this difference in mortality between sexes was only found in patients younger than 45 years. Our study also suggests that one possible contributor to the higher survival rates in females could be the differences in the occurrence of life-threatening complications. However, other potential factors such as social status and living habits cannot be ruled out without further study.
